# Perceptions, Usage, and Educational Impact of ChatGPT Among Medical Students in Germany: Cross-Sectional Mixed Methods Survey

**DOI:** 10.2196/81484

**Published:** 2025-11-11

**Authors:** Anna Fußhöller, Fabian Lechner, Nadine Schlicker, Felix Muehlensiepen, Andreas Mayr, Sebastian Kuhn, Martin Christian Hirsch, Johannes Knitza

**Affiliations:** 1 Institute for Digital Medicine, School of Medicine, Philipps-Universität Marburg, Baldingerstrasse 1, Marburg, 35043, Germany, 49 (0)6421586258; 2 Institute for Artificial Intelligence in Medicine, School of Medicine, Philipps-Universität Marburg, Marburg, Germany; 3 Center for Health Services Research, Brandenburg Medical School Theodor Fontane, Rüdersdorf, Germany; 4 Department of Cardiology, Angiology and Intensive Care Medicine, Deutsches Herzzentrum der Charité, Berlin, Germany; 5 Institute for Medical Biometry and Statistics, Philipps-Universität Marburg, Marburg, Germany

**Keywords:** medical education, artificial intelligence, ChatGPT, large language model, digital health literacy, medical students, Germany, survey, self-directed learning

## Abstract

**Background:**

Large language models such as ChatGPT offer significant opportunities for medical education. However, empirical data on actual usage patterns, perceived benefits, and limitations among medical students remain limited.

**Objective:**

This study aimed to assess how medical students in Germany use ChatGPT, their perceptions of its educational value, and the challenges and concerns associated with its use.

**Methods:**

A cross-sectional 17-item online survey was conducted between May and August 2024 among medical students from Philipps University Marburg, Germany. A mixed methods approach was applied, combining descriptive and inferential statistical analysis with qualitative content analysis of open-ended responses.

**Results:**

A total of 84 fully completed surveys were included in the analysis (response rate: 26.7%; 315 surveys started). Overall, 76.2% (64/84) of the participants reported having used ChatGPT for medical education, with significantly higher usage during exam periods (*P*=.003). Preclinical students reported higher overall usage than clinical students (*P*=.02). ChatGPT was primarily used for summarizing information by 60.7% (51/84) of students, for literature research by 57.7% (49/84), and for clarifying concepts by 47.1% (40/84). A total of 70.2% (59/84) felt that it helped them save time, and 51.2% (43/84) reported an improved understanding of content. In contrast, only 31% (26/84) saw benefits for applying knowledge and 15.5% (13/84) for long-term knowledge retention. Qualitative responses highlighted clear benefits such as time savings and support in exam preparation, while also pointing to potential applications in clinical documentation and expressing concerns about misinformation and source transparency. However, 73.3% (55/75) expressed concerns about misinformation, and 72.6% (61/84) reported lacking confidence in their artificial intelligence (AI)–related skills. Only 41.7% (35/84) stated that they trust ChatGPT’s outputs. Students who used the tool more frequently also reported higher levels of trust in ChatGPT’s outputs (*r*=0.374, *P<*.001). Over 70% of respondents indicated a strong desire for increased integration of AI-related education and practical applications within the medical curriculum.

**Conclusions:**

ChatGPT was already widely used among medical students, especially in exam preparation and the early stages of training. Students valued its efficiency and support for understanding complex material, but its long-term influence on learning is limited. Concerns about reliability, source transparency, and data privacy remain, and AI skills played a key role in shaping usage. These findings underscore the need to integrate structured, practice-oriented AI education into medical training to support critical, informed, and ethical use of large language models.

## Introduction

The integration of large language models (LLMs) into higher education is rapidly transforming teaching and learning processes, particularly in the field of medicine [1-3]. These models are capable of generating human-like text, answering complex questions, and simulating interactive dialogue, which makes them uniquely suited for educational settings [4]. Among the most prominent artificial intelligence (AI) tools is ChatGPT [5]. Since its public release in November 2022, ChatGPT has become one of the most widely adopted AI tools worldwide, with millions of users, including a growing number of medical students [6,7].

LLMs offer medical students new opportunities for self-directed learning. These include simplifying dense academic texts, generating practice questions, simulating patient interactions, and providing personalized feedback. Early evidence suggests that these functionalities can support knowledge acquisition, comprehension, and problem-solving skills in medical education [8]. ChatGPT’s ability to mimic personalized tutoring environments, generate differential diagnoses based on input data, and simulate realistic clinical scenarios presents a novel form of digital support for students’ learning processes [1].

Nevertheless, ChatGPT has certain restrictions. ChatGPT can produce misleading or incorrect content while presenting it with convincing linguistic fluency, lacks source transparency, and does not account for evidence hierarchies in medical knowledge [9-11]. Privacy concerns are also relevant, as user inputs are processed on external servers, potentially compromising patient confidentiality when clinical case data are entered [12]. Furthermore, excessive reliance on AI tools may hinder the development of critical thinking skills [2,9] and potentially lead to suboptimal clinical decision-making [13].

Although interest in the educational potential of LLMs is increasing, evidence on their use and impact among medical students remains limited [14]. This study addressed this gap by examining how medical students in Germany use ChatGPT, their perceptions of its educational value, and the challenges and concerns associated with its use.

## Methods

### Study Design

The survey encompassed a total of 17 questions (Multimedia Appendix 1) and was reported in accordance with the CHERRIES (Checklist for Reporting Results of Internet E-Surveys) checklist [15]. The web-based survey was conducted via SoSciSurvey between May and August 2024. Medical students from University Marburg were recruited through student mailing lists, medical lectures at Philipps University Marburg, and by personal invitation from AF. The open survey was developed based on a review of related literature [16-18]. Adaptive questioning was applied to tailor the survey experience and reduce participant burden. Participants who indicated using other educational software or AI tools were asked to specify which ones. The following open-ended questions were included: “What concerns do you have when using ChatGPT?” “In which areas do you see potential in using ChatGPT?” “For which subjects or areas do you primarily use ChatGPT and why?” The survey was pretested for usability and technical functionality by 3 medical students who were not part of the final sample. Their feedback was used to refine the wording, layout, and navigation of the questionnaire before launching the final version via SoSciSurvey.

### Ethical Considerations

The study was reviewed by the Ethics Committee of Philipps University Marburg, which granted an exemption waiver and raised no objections (reference number 24‐80 ANZ). All participants received an information sheet before beginning the survey and provided electronic informed consent; participation was voluntary and could be discontinued at any time. Data were collected anonymously, with no personal identifiers recorded, and open-text responses were screened to ensure confidentiality. No financial or nonfinancial incentives were offered.

### Data Analysis

A mixed methods approach was applied, combining quantitative and qualitative data through both closed and open-ended survey questions. This design allows for the identification of statistical patterns while also capturing insights into individual experiences.

Quantitative data were analyzed using jamovi (version 2.3.28.0; The Jamovi Project) and Microsoft Excel (version 16.89.1; Microsoft Corp) to compute descriptive statistics and explore relationships between variables. For group comparisons of ordinal or nonnormally distributed variables, the Mann-Whitney *U* test or the Welch *t* test (in cases of variance inhomogeneity) was applied. Paired comparisons between two related conditions (eg, semester vs exam period) were analyzed using the Wilcoxon signed-rank test. Correlations were assessed using Pearson correlation coefficient and Spearman rho, where appropriate. Differences in proportions were tested using binomial tests. Statistical significance was defined as *P*<.05.

Qualitative responses were analyzed using *MAXQDA* (VERBI Software). An inductive approach was applied to structure the open-ended answers. The data were systematically coded by one researcher who developed the main codes (eg, barriers, advantages) and subcodes (eg, time-saving or lack of trust), which were used to identify recurring themes and patterns. Based on these thematic categories, frequency tables were generated to quantify the distribution of responses and support interpretive conclusions. Representative quotations were then extracted to illustrate key points within each category.

Medical education in Germany is divided into 2 phases. First, there is a preclinical phase that lasts 2 years and focuses on foundational subjects and conveys more theoretical knowledge. After passing the “Physikum,” students enter the clinical phase, which lasts 4 years and provides more practical and patient-centered training. An exploratory subgroup analysis was performed based on this distinction.

## Results

### Study Population

A total of 315 medical students from German universities participated in the survey, of whom 26.7% (84/315) provided fully completed surveys that were included in the final analysis. Among these, 69% (58/84) were female. The mean age of participants was 23.7 years (median 23; range 19‐30), and the average semester of study was 5.5 (SD 2.2). Of the participants, 22 were enrolled in the preclinical phase of medical education and 62 in the clinical phase.

### ChatGPT Usage

Of all participants, 76.2% (64/84) stated that they have used ChatGPT for medical education purposes. The highest proportion of students used it once per week(Multimedia Appendix 2). A Wilcoxon signed-rank test indicated that students used ChatGPT significantly more often during exam preparation periods than during regular semester periods (*P*=.003).

Seventy-five percent (63/84) of the students reported not using any other AI tools for studying besides ChatGPT. DeepL was the most frequently used other AI tool, reported by 38.9% (7/18) of students who used another tool. In addition, a Mann-Whitney *U* test showed that preclinical students (mean 2.82) reported significantly higher overall ChatGPT usage than clinical students (mean 2.20; *P*=.02). For example, a preclinical student stated, “In biochemistry and anatomy, it provides faster answers than looking things up in textbooks for general questions.” No significant differences in usage were found concerning age or gender. ChatGPT was used for various different academic tasks. We found that 60.7% (51/84) of respondents claimed to use ChatGPT for summarization, 57.7% (49/84) for research and literature study, and 47.6% (40/84) to clarify questions and explain subject-specific concepts (Multimedia Appendix 3).

ChatGPT was also reported to be used across various subjects within the medical curriculum. The most frequently mentioned area was general medical studies, cited by 37.5% (18/48) of students, followed by medical history and ethics with 27.1% (13/48), and biochemistry with 14.6% (7/48). Physiology was mentioned in 12.5% (6/48) of responses and anatomy and histology in 10.4% (5/48). Less frequently named subjects included pharmacology, mentioned by 8.3% (4/48) of students, biology, cited by 6.3% (3/48), and both chemistry and physics, with 4.2% (2/48) each.

### Perceived Impact of ChatGPT

**Figure 1.**
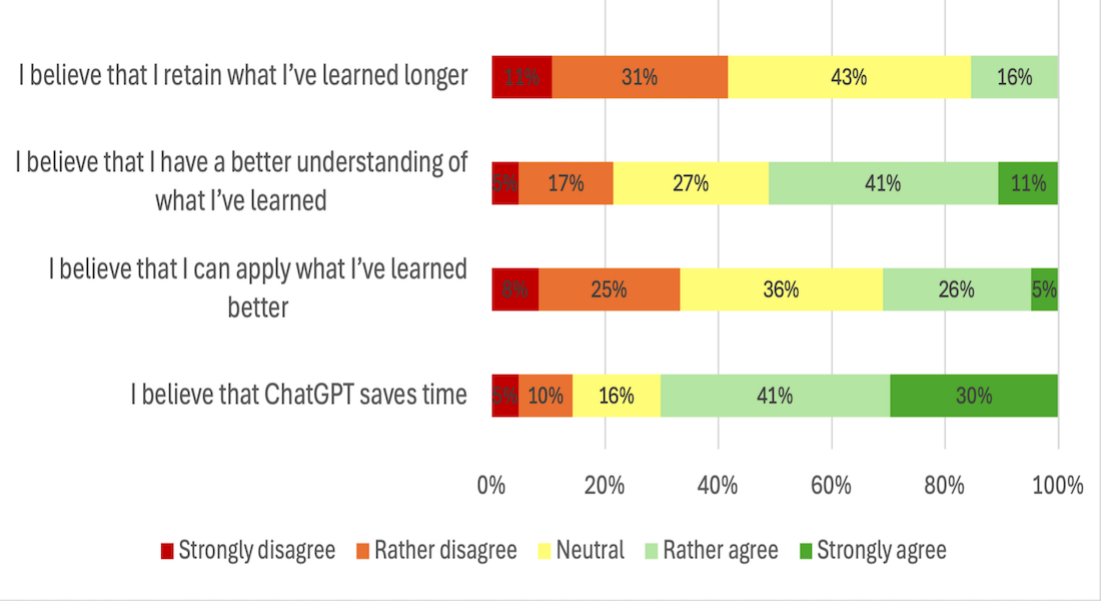
Perceived impact of ChatGPT on learning efficiency, understanding, application, and knowledge retention among medical students.

The majority (59/84, 70.2%) of students agreed that using ChatGPT helped them save time (Figure 1). One student stated, “It helps to condense complex content into short summaries that focus on the essentials, making it easier to get started. I often spend a long time searching textbooks for unclear terms, and ChatGPT can often provide helpful insights or even completely resolve my question.” Furthermore, 51.2% (43/84) reported improved understanding of the content, while 31% (26/84) felt that it enhanced their ability to apply what they had learned. Only 15.5% (13/84) agreed that ChatGPT improved their long-term information retention.

### Perceived Benefits and Trust

In all, 38.1% (32/84) stated that ChatGPT had advantages over other learning platforms. The advantages listed (optional answer) were mostly more specific and individual benefits, reported by 50% (10/20) of respondents; time saving, mentioned by 35% (7/20); and more comprehensible explanations, cited by 15% (3/20). One student explained that “targeted questions can be answered within seconds—questions that might not be addressed in some books or would otherwise require extensive research.” Furthermore, 41.7% (35/84) of the students agreed that they trust the results provided by ChatGPT. A significant positive correlation was found between trust and usage frequency (Pearson *r*=0.374, *P<*.001).

### Potential and Barriers

Students were invited to suggest areas in which they could see potential for using ChatGPT in a free-text answer. The most frequently mentioned areas included explanation, named by 22.5% (20/89) of students; summarization, mentioned by 18% (16/89); support with everyday hospital tasks such as medical reports, cited by 15.7% (14/89); text processing, mentioned by 14.6% (13/89); time saving, reported by 12.4% (11/89); research, indicated by 11.2% (10/89); and use as a learning tool, stated by 5.6% (5/89).

In another open-ended question, students were asked to share their concerns about using ChatGPT. The majority, 73.3% (55/75), expressed worries about incorrect or inaccurate information, as exemplified by this quote: “I don’t know how trustworthy the sources used are.” In addition, 16% (12/75) of the students mentioned concerns about unreliable or nonexistent sources, 6.7% (5/75) claimed a potential decline in academic independence, and 4% (3/75) were concerned about the handling of personal data. One student stated the following fear: “That I rely too much on a system, which may mean I retain less myself.”

### AI Knowledge

Most students reported becoming aware of ChatGPT through personal technical interest (44/84, 52.4%) or media coverage (43/84, 51.2%). Recommendations from others were also a significant factor, reported by 45.2% (38/84). Additionally, 16.7% (14/84) reported discovering ChatGPT through independent internet research, while 13.1% (11/84) mentioned social media. Despite this widespread awareness, the majority of students indicated a lack of deeper understanding: 72.6% (61/84) of the students stated that they are not well-acquainted with AI and its possibilities (Multimedia Appendix 4).

**Figure 2.**
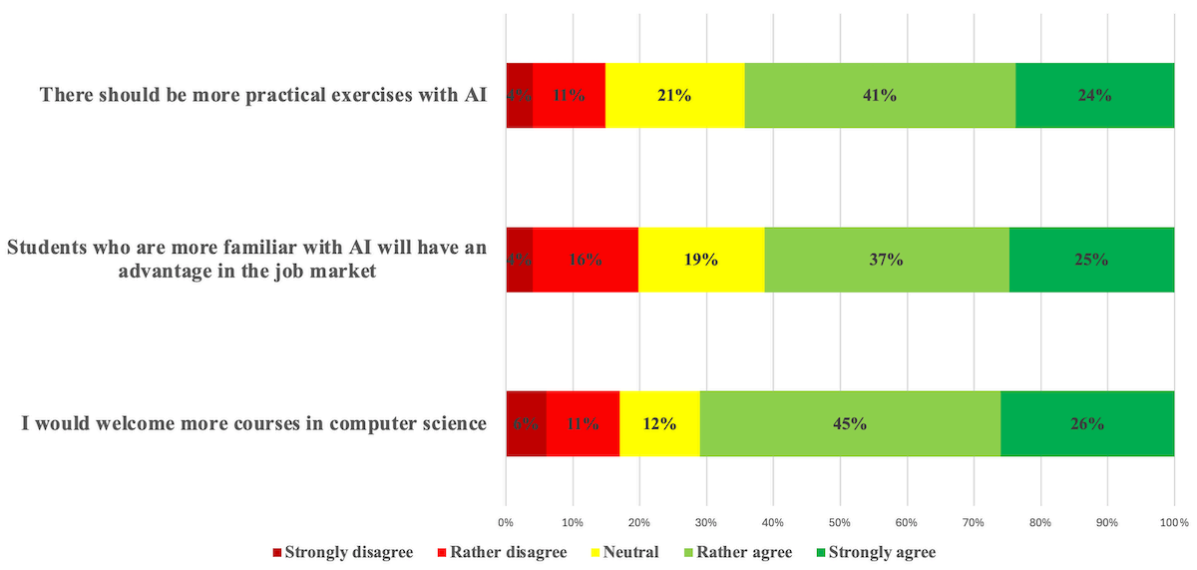
Students’ perspectives on computer science and AI in their education and career. AI: artificial intelligence.

Most of the students (60/84, 71.4%) would appreciate more courses in computer science in order to learn more about the possibilities of AI in medicine, and 74.3% (54/84) of the students would appreciate more practical exercises with AI. In addition, 61.9% (52/84) agreed that students more familiar with AI have a career advantage (Figure 2).

## Discussion

### Principal Findings

This study found that ChatGPT was widely used among German medical students, particularly during exam periods and in the preclinical phase. Students reported using it mainly for summarizing material, literature searches, and clarification of concepts. Although many students valued its efficiency and perceived improvements in comprehension, fewer saw benefits for application or long-term retention. Concerns about misinformation and source credibility were common, and AI literacy strongly influenced trust and frequency of use. The majority of medical students reported low confidence in their AI skills and expressed a strong interest in additional AI training. Students’ perceptions of ChatGPT appear to reflect broader attitudes toward digital learning technologies in general, rather than being unique to LLMs and ChatGPT.

### LLM Usage

The large proportion of medical students (76.2%) who had already used ChatGPT to support their studies aligns with recent international data: for instance, a 2025 survey conducted in Egypt reported a similarly high usage rate of 78.5% among students [19], and a 2024 survey from China reported a usage rate of 62.9% [20]. In contrast, an earlier US-based study from 2024 documented a considerably lower usage rate of only 48.9% [21], and a survey across Germany, Austria, and Switzerland, published in 2024, revealed a rate of 38.8% [16]. These differences are likely attributable to the varying time points of data collection and suggest a growing trend in ChatGPT adoption among medical students over time.

To the best of our knowledge, this is the first study to demonstrate a significantly increased use of ChatGPT during examination periods of medical students. A Spanish study from 2024 among nursing students supports this observed trend. The majority of students considered ChatGPT to be useful or very useful for exam preparation, and 89% reported that it enhanced their academic performance to some extent [22]. These findings underscore the growing relevance of LLMs, particularly during high-stress periods such as exams, when students seek efficient and effective study support.

We found that preclinical students (first 2 years of medical school in Germany) reported significantly higher overall usage than clinical students. Similar results were observed in a 2024 US study, which reported significantly higher usage of ChatGPT among first-year medical students [23]. This pattern may reflect the stronger focus on theoretical content and self-directed learning in the early years of medical education, where tools like ChatGPT are particularly helpful for summarizing complex material or clarifying foundational concepts. In contrast, a study among undergraduate health care students in Malaysia from 2024 found opposite results. They state that fifth-year students were significantly more likely to use ChatGPT for academic purposes than first-year students [24]. This discrepancy could be due to differences in curricular structure, teaching methods, or digital tool integration. For instance, senior students in some contexts may face more complex clinical case studies and written assignments, prompting them to seek AI-based support for academic writing or clinical reasoning tasks.

Our findings indicate that students use ChatGPT in diverse ways. This corresponds in part with findings from a US-based study, where 59.6% of students used ChatGPT for proofreading and grammar correction. Notably, the majority of respondents in that study (89.9%) reported that ChatGPT enhanced their research productivity, which may reflect a broader trend toward the use of AI tools to support academic work [23].

Free-text responses suggested that students already envisioned potential future applications of ChatGPT in both clinical and academic settings. Most frequently, they emphasized its value in explaining medical content, summarizing information, and supporting routine clinical tasks such as drafting medical reports. These perceptions closely align with findings from a US study, where 22.5% of students who had completed clinical rotations reported using ChatGPT during their clerkship. Among these students, the tool was primarily used to gather information on diagnoses, pharmacology, and patient care planning, as well as to assist with writing clinical notes [23].

### Perceived LLM Advantages and Barriers

Our findings showed that roughly one-third of the students considered ChatGPT to offer advantages over other learning platforms. A US study further reported that some students preferred using ChatGPT over consulting professors (45.3%), using textbooks (42.2%), or attending lectures (31.7%) [21]. The authors attribute this preference to ChatGPT’s ability to deliver instant, personalized, and user-friendly responses, which aligns with our findings. A recent meta-analysis highlighted the advantages of ChatGPT use, such as improved academic performance and a reduction in mental effort [25]. However, a complementary review has raised concerns about the potential for “never-skilling,” whereby overreliance on LLMs may hinder skill acquisition [2]. To address this challenge, the authors introduced the DEFT-AI (Diagnosis, Evidence, Feedback, and Teaching) framework, which emphasizes fostering critical thinking as a safeguard against such risks. A subsequent review concluded that the acquisition of theoretical knowledge was comparable between generative AI–based instruction and traditional teaching methods in medical education [26].

Our findings suggest that while ChatGPT is perceived as a valuable support tool, particularly for enhancing efficiency and comprehension, its influence on long-term knowledge retention appears to be limited. Marton and Säljö’s concept of *approaches to learning* [27] offers further insight: if ChatGPT is used to engage with underlying concepts and to seek understanding, it may enhance comprehension. However, when used primarily to complete tasks quickly, as part of a surface approach, its educational benefit is likely to be minimal. The combination of high reported time-saving and low reported gains in knowledge application and retention in our data suggests that both usage patterns coexist. This highlights the need to promote critical engagement with AI tools and to provide educational frameworks that encourage responsible and reflective use.

When evaluating students’ trust in ChatGPT, the results revealed a rather cautious stance. This finding is consistent with previous studies, which reported that approximately half of the students trusted ChatGPT’s responses [19,23]. Interestingly, in our study, there was a significant positive correlation between trust and usage frequency. In the study of Malaysian health care students, higher knowledge and positive attitudes correlated with increased academic usage [24]. Taken together, these findings suggest that students’ engagement with AI tools is not solely driven by external factors such as availability or technical ease of use, but also by internal factors like trust, familiarity, and perceived value. This highlights the importance of building digital literacy and user confidence to foster responsible and effective use.

In this study, students also voiced critical concerns regarding the use of ChatGPT. These concerns reflect broader criticisms discussed in the literature, particularly regarding the risk of misinformation, facilitated plagiarism [20], lack of source transparency, and the impact of AI tools on core academic competencies [24,28,29].

### AI Knowledge and Education

Most medical students lacked confidence in their AI skills and expressed a desire for more AI training. These results emphasize the need to integrate structured and practice-oriented AI education into the medical curriculum and mirror results from previous studies [28-31]. A recently evaluated AI module for German medical students received a 100% peer recommendation rate, reflecting high levels of acceptance and perceived value [32]. Furthermore, a blended-learning course by Thomae et al [31] integrated ChatGPT into core teaching units and emphasized student reflection and AI output evaluation, improving both engagement and perceived relevance. A previous survey also highlighted that hands-on AI experience was significantly linked to more positive perceptions of its role in improving patient care [17]. To build on these early models, future research should prioritize intervention studies that assess learning outcomes. A significant barrier to the implementation of AI education may be the limited digital competence among educators. The Check-In tool, consisting of 22 items derived from the European Framework for the Digital Competence of Educators (DigCompEdu), provides a structured approach to assessing current competencies and systematically identifying areas for improvement [3].

### Limitations

This study is subject to several limitations. Our findings should be viewed as a snapshot of early ChatGPT adoption, which may not fully capture current practices due to rapid AI developments. Furthermore, data collection was based on a voluntary online questionnaire. This may have introduced a self-selection bias, as students with an interest in technology may have been more likely to participate. Additionally, reliance on self-reported measures may introduce inaccuracies due to factors such as social desirability and recall bias. As a result, the reported ChatGPT usage in this study could be overestimated compared to participants’ actual average usage. Although 315 students accessed the survey, only 84 provided complete responses, which reflects the generally low response rates commonly observed in medical education online surveys [33]. This relatively low completion rate may be attributed to several factors, including the voluntary nature of participation, the overall length of the questionnaire, and the inclusion of open-ended questions, which may have required more time and cognitive effort. A further limitation is that the analysis of open-ended responses was conducted by a single researcher, which may have introduced subjectivity and limited intercoder reliability. Although this approach ensured consistency in coding, future studies would benefit from involving multiple coders and formal reliability checks to strengthen the robustness of qualitative findings.

### Conclusions

ChatGPT was widely used by medical students in Germany, particularly during exam preparation and in the preclinical phase of training. Students primarily valued the tool for its efficiency, summarization capabilities, and ability to support understanding of complex material. Key concerns included misinformation, lack of source transparency, and data privacy risks, all of which contribute to variable levels of student trust. Notably, the strong demand for AI-related training underscores the urgency of integrating structured, practice-oriented AI education into medical curricula. As LLMs become increasingly prevalent in health care and education, promoting critical, informed, and ethically responsible use among future clinicians is essential.

## Supplementary material

10.2196/81484 Multimedia Appendix 1 Original questionnaire and English translation.

10.2196/81484 Multimedia Appendix 2 ChatGPT usage among medical students during the semester and exam period.

10.2196/81484 Multimedia Appendix 3 Frequency of ChatGPT usage across different academic tasks among medical students.

10.2196/81484 Multimedia Appendix 4 Proportions of students indicating how acquainted they are with artificial intelligence and its possibilities.

## Abbreviations

AI: artificial intelligence
CHERRIES: Checklist for Reporting Results of Internet E-Surveys
LLM: large language model
