# Supplementary material for: Perceptions, Usage, and Educational Impact of ChatGPT Among Medical Students in Germany: Cross-Sectional Mixed Methods Survey
Source: JMIR Form Res. 2025 Nov 11;9:e81484. doi: 10.2196/81484 (PMC12604828; doi:10.2196/81484)
Supplement: Multimedia Appendix 1 [file formative-v9-e81484-s001.pdf]

Seite 01

Hinweis

Seite **1** des Fragebogens **base** enthält weder Fragen noch andere Elemente. Öffnen Sie diesen Fragebogen unter "Fragebogen zusammenstellen" zum Bearbeiten und ziehen Sie Fragen oder andere Elemente aus der Auswahlliste (rechts) in die Fragebogenseite.

Seite 02

1. Wie alt sind Sie?

SD02

Ich bin ☐ Jahre alt

2. Welches Geschlecht haben Sie?

SD01

- ○ weiblich
- ○ männlich
- ○ divers

3. In welchem Semester sind Sie?

SE01

Ich bin im ☐ Semester

4. Wie oft nutzen Sie ChatGPT ..

FR01

.. während des Semesters?

Ich benutze es nicht	0-1 Mal pro Woche	2-5 Mal pro Woche	6-10 Mal pro Woche	Ich benutze es häufiger	*kann ich nicht beurteilen*

.. während der Klausurenphase?

Ich benutze es nicht	0-1 Mal pro Woche	2-5 Mal pro Woche	6-10 Mal pro Woche	Ich benutze es häufiger	*kann ich nicht beurteilen*

Seite 04

5. Wie sehr vertrauen Sie den Aussagen von ChatGPT?

VE01

Ich vertraue den Aussagen von ChatGPT

stimme gar nicht zu	stimme eher nicht zu	unent-schieden	stimme eher zu	stimme voll zu	*kann ich nicht beurteilen*

EF01

6. Hat sich die Effektivität Ihres Lernens durch die Nutzung von ChatGPT verändert?

Ich habe das Gefühl, mit ChatGPT Zeit zu sparen

stimme gar nicht zu	stimme eher nicht zu	unent- schieden	stimme eher zu	stimme voll zu	*kann ich nicht beurteilen*

Ich habe das Gefühl, das Gelernte besser anwenden zu können

stimme gar nicht zu	stimme eher nicht zu	unent- schieden	stimme eher zu	stimme voll zu	*kann ich nicht beurteilen*

Ich habe das Gefühl, das Gelernte besser verstanden zu haben

stimme gar nicht zu	stimme eher nicht zu	unent- schieden	stimme eher zu	stimme voll zu	*kann ich nicht beurteilen*

Ich habe das Gefühl, das Gelernte länger zu behalten

stimme gar nicht zu	stimme eher nicht zu	unent- schieden	stimme eher zu	stimme voll zu	*kann ich nicht beurteilen*

Seite 06

NU02

7. Im Rahmen meines Studiums nutze ich KI..

- ☐ ..für Recherchen und Literaturstudium
- ☐ ..zur Textzusammenfassung
- ☐ ..zur Klärung von Verständnisfragen und um mir fachspezifische Konzepte erklären zu lassen
- ☐ ..zur Textanalyse, Textverarbeitung, Texterstellung
- ☐ ..zur Prüfungsvorbereitung
- ☐ ..um mir Übungsfragen generieren zu lassen
- ☐ Sonstige: ________

Seite 07

BE01

8. Welche Bedenken haben Sie bei der Nutzung von ChatGPT ?

Bitte schreiben Sie Ihre Gedanken in das Feld

Seite 08

PO01

9. In welchen Bereichen sehen Sie Potenziale bei der Nutzung von ChatGPT ?

Bitte schreiben Sie Ihre Gedanken in das Feld

Seite 09

MO01

10. Würden Sie gerne mehr über die Möglichkeiten von KI in der Medizin lernen?

Würden Sie mehr Lehrveranstaltungen, im Bereich Informatik, befürworten?

stimme gar nicht zu	stimme eher nicht zu	unent-schieden	stimme eher zu	stimme voll zu	*kann ich nicht beurteilen*

Sollte es Ihrer Meinung nach mehr praktische Übungen mit KI geben?

stimme gar nicht zu	stimme eher nicht zu	unent-schieden	stimme eher zu	stimme voll zu	*kann ich nicht beurteilen*

Denken Sie, dass Studierende die sich besser mit KI auskennen, später einen Vorteil in der Arbeitswelt haben?

stimme gar nicht zu	stimme eher nicht zu	unent-schieden	stimme eher zu	stimme voll zu	*kann ich nicht beurteilen*

Seite 10

MI01

11. Ich kenne mich gut mit KI und dessen Möglichkeiten aus

Wie schätzen Sie sich selbst ein?

stimme gar nicht zu	stimme eher nicht zu	unent-schieden	stimme eher zu	stimme voll zu	*kann ich nicht beurteilen*

Seite 11

AF01

12. Wie sind Sie auf ChatGPT aufmerksam geworden?

- ☐ Internetrecherche
- ☐ Empfehlungen
- ☐ Medienberichte
- ☐ Soziale Medien
- ☐ Technisches Interesse
- ☐ Sonstiges: ________

Seite 12

FA01

13. Für welche Fächer/Fachbereiche nutzen Sie ChatGPT vorwiegend und weswegen?

Bitte schreiben Sie Ihre Gedanken in das Feld

____

VO01

14. Sehen sie einen Vorteil bei der Nutzung von ChatGPT, im Gegensatz zu anderen Lernplattformen?

- Ja
- Nein
- Weiß ich nicht

1 aktive(r) Filter

Filter VO01/F1
Wenn eine der folgenden Antwortoption(en) ausgewählt wurde: **1**
Dann Frage/Text **VO02** später im Fragebogen anzeigen (sonst ausblenden)

VO02

15. Falls Ja, bei welcher Lernplattform und inwiefern ?

Bitte schreiben Sie Ihre Gedanken in das Feld

16. Nutzen Sie, außer ChatGPT, noch andere KI Tools zum lernen?

TO01

- ◯ Ja
- ◯ Nein
- ◯ Weiß ich nicht

1 aktive(r) Filter

Filter TO01/F1
Wenn eine der folgenden Antwortoption(en) ausgewählt wurde: **1**
Dann Frage/Text **TO02** später im Fragebogen anzeigen (sonst ausblenden)

17. Falls Ja, Welche Tools nutzen Sie?

TO02

Bitte schreiben Sie Ihre Gedanken in das Feld

Vielen Dank für Ihre Teilnahme!

Wir möchten uns ganz herzlich für Ihre Mithilfe bedanken.

Ihre Antworten wurden gespeichert, Sie können das Browser-Fenster nun schließen.

Möchten Sie in Zukunft an interessanten und spannenden Online-Befragungen teilnehmen?

Wir würden uns sehr freuen, wenn Sie Ihre E-Mail-Adresse für das SoSci Panel anmelden und damit wissenschaftliche Forschungsprojekte unterstützen.

E-Mail: ______ **Am Panel teilnehmen**

Die Teilnahme am SoSci Panel ist freiwillig, unverbindlich und kann jederzeit widerrufen werden.
Das SoSci Panel speichert Ihre E-Mail-Adresse nicht ohne Ihr Einverständnis, sendet Ihnen keine Werbung und gibt Ihre E-Mail-Adresse nicht an Dritte weiter.

Sie können das Browserfenster selbstverständlich auch schließen, ohne am SoSci Panel teilzunehmen.

Anna Fußhöller, Philipps-Universität Marburg – 2024

English translation of the survey

1. How old are you?

    - I am ___ years old.

2. What is your gender?

    - Female
    - Male
    - Diverse

3. What semester are you in?

    - I am in the ___ semester.

4. How often do you use ChatGPT..

    ..during the semester?

    ..during exam period?

    • I don't use it

    • 0–1 times per week

    • 2–5 times per week

    • 6–10 times per week

    • I use it more often

    • Cannot judge

5. How much do you trust the statements made by ChatGPT?

    I trust the statements made by ChatGPT

    • Strongly disagree

- Rather disagree
- Neutral
- Rather agree
- Strongly agree
- Cannot judge

6. Has the effectiveness of your learning changed through the use of ChatGPT?

- I feel that ChatGPT saves time
- I feel that I can apply what I've learned better
- I feel that I understand what I've learned better
- I feel that I retain what I've learned longer

Scale for all: Strongly disagree to Strongly agree + Cannot judge

7. In the context of my studies, I use AI for... (multiple selection possible)

- Research and literature review
- Summarizing texts
- Clarifying understanding and explaining subject-specific concepts
- Text analysis, processing, and generation
- Exam preparation
- Generating practice questions
- Other: ___

8. What concerns do you have when using ChatGPT?

(Open text field)

9. In which areas do you see potential in using ChatGPT?

(Open text field)

10. Would you like to learn more about the possibilities of AI in medicine?

- Would you welcome more courses in computer science?
- Do you think there should be more practical exercises with AI?
- Do you think that students who are more familiar with AI will have an advantage in the job market?

Scale for all: Strongly disagree to Strongly agree + Cannot judge

11. I am well versed with AI and its possibilities

- Strongly disagree
- Rather disagree
- Neutral
- Rather agree
- Strongly agree
- Cannot judge

12. How did you become aware of ChatGPT? (multiple selection possible)

- Internet search
- Recommendations
- Media reports
- Social media
- Technical interest
- Other: ___

13. For which subjects or areas do you primarily use ChatGPT and why?

(Open text field)

14. Do you see an advantage in using ChatGPT compared to other learning platforms?

- Yes

- No
- I don't know

15. If yes, with which platform and in what way?

    (Open text field)

16. Apart from ChatGPT, do you use other AI tools for learning?

- Yes
- No
- I don't know

17. If yes, which tools do you use?

    (Open text field)
