# Supplementary figures and images for: Perceptions, Usage, and Educational Impact of ChatGPT Among Medical Students in Germany: Cross-Sectional Mixed Methods Survey
Source: JMIR Form Res. 2025 Nov 11;9:e81484. doi: 10.2196/81484 (PMC12604828; doi:10.2196/81484)

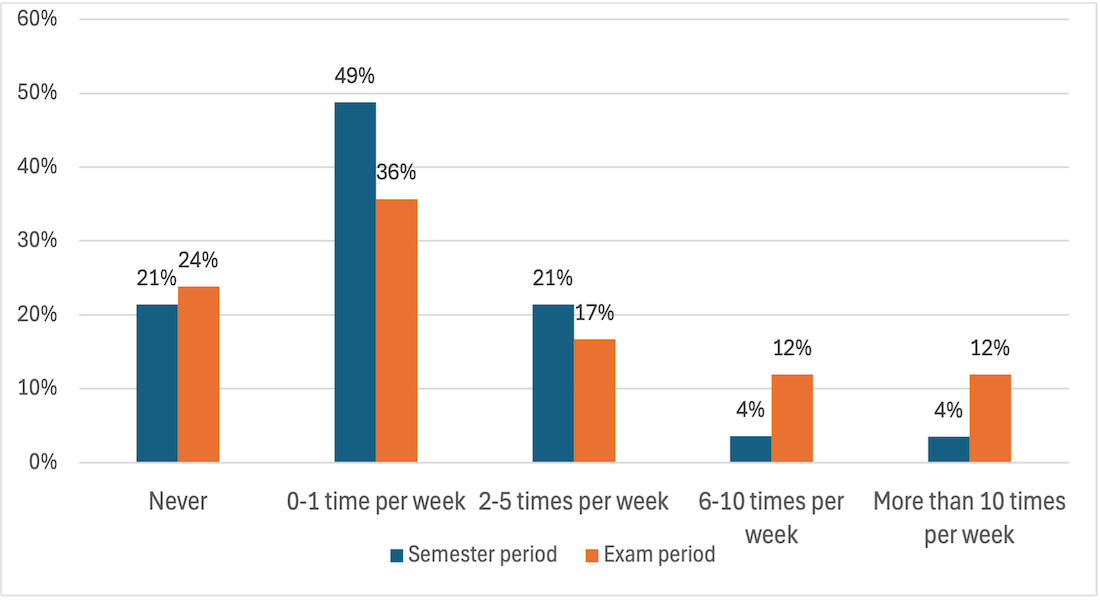

Supplement: Multimedia Appendix 2 [file formative-v9-e81484-s002.png]

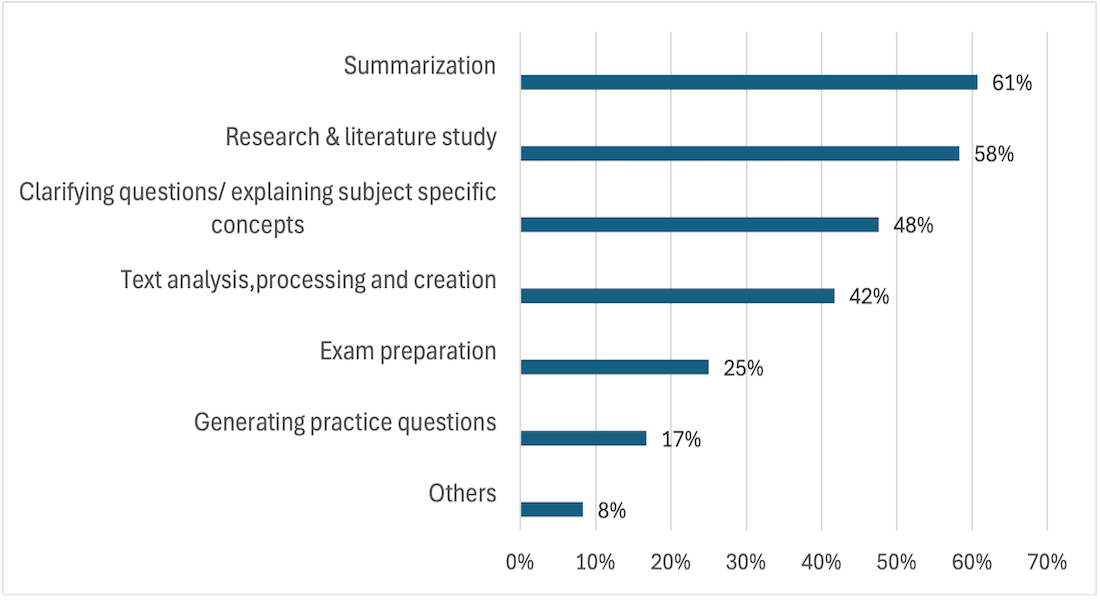

Supplement: Multimedia Appendix 3 [file formative-v9-e81484-s003.png]

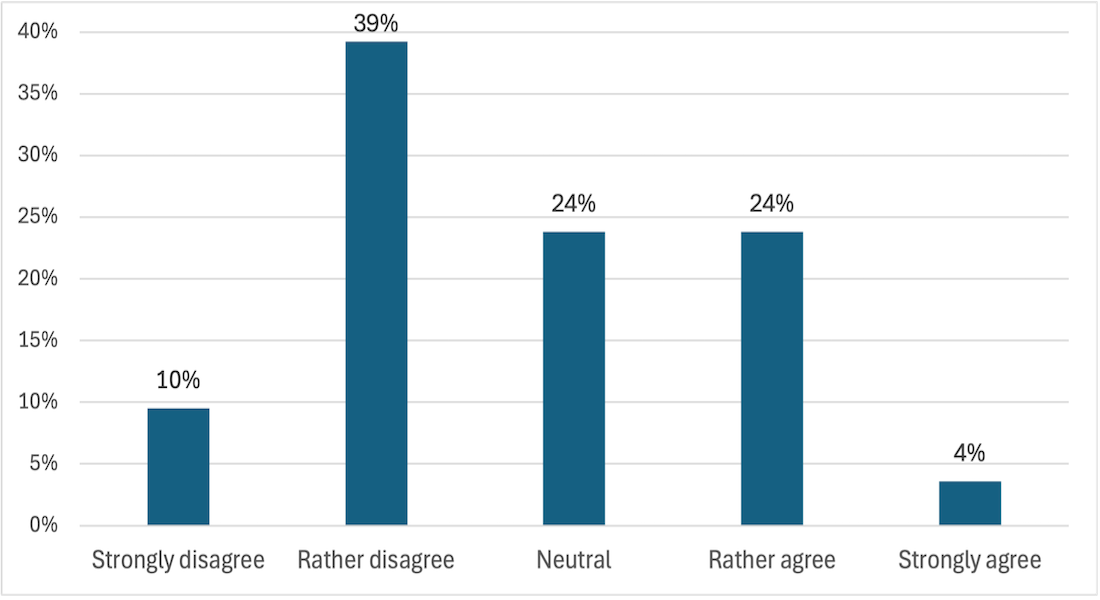

Supplement: Multimedia Appendix 4 [file formative-v9-e81484-s004.png]
